# Potential health benefits of *Nigella sativa* on diabetes mellitus and its complications: A review from laboratory studies to clinical trials

**DOI:** 10.3389/fnut.2022.1057825

**Published:** 2022-11-10

**Authors:** Siti Hajar Adam, Noor Mohd Nasri, Mohd Izhar Ariff Mohd Kashim, Erny Haslina Abd Latib, Muhammad Amirul Aiman Ahmad Juhari, Mohd Helmy Mokhtar

**Affiliations:** ^1^Preclinical Department, Faculty of Medicine and Defence Health, Universiti Pertahanan Nasional Malaysia, Kuala Lumpur, Malaysia; ^2^Department of Nursing, Faculty of Medicine, Universiti Kebangsaan Malaysia, Kuala Lumpur, Malaysia; ^3^Department of Physiology, Faculty of Medicine, Universiti Kebangsaan Malaysia, Kuala Lumpur, Malaysia; ^4^Centre of Shariah, Faculty of Islamic Studies, Universiti Kebangsaan Malaysia, Bangi,Selangor, Malaysia; ^5^Insitute of Islam Hadhari, Universiti Kebangsaan Malaysia, Bangi, Selangor, Malaysia; ^6^Bioaromatic Research Centre, Universiti Malaysia Pahang, Gambang, Pahang, Malaysia; ^7^Faculty of Forestry and Environment, Universiti Putra Malaysia, Serdang, Selangor, Malaysia

**Keywords:** *Nigella sativa*, thymoquinone, diabetes mellitus, antidiabetic, antioxidative, hypoglycaemic

## Abstract

This review aims to gather and summarize up-to-date information on the potential health benefits of *Nigella sativa* (NS) on diabetes mellitus (DM) and its complications from different animal models, clinical trials and *in vitro* studies. DM is one of the most prevalent metabolic disorders resulting from chronic hyperglycaemia due to problems in insulin secretion, insulin action or both. It affects people regardless of age, gender and race. The main consequence of DM development is the metabolic dysregulation of glucose homeostasis. Current treatments for DM include pharmacological therapy, insulin and diabetic therapy targeting β cells. Some of these therapeutic approaches are promising; however, their safety and effectiveness remain elusive. Since ancient times, medicinal plants have been used and proven effective against diseases. These plants are believed to be effective and benefit physiological and pathological processes, as they can be used to prevent, reduce or treat multiple diseases. *Nigella sativa* Linn. is an annual indigenous herbaceous plant belonging to Ranunculaceae, the buttercup family. NS exhibits multifactorial activities; it could ameliorate oxidative, inflammatory, apoptotic and insulinotropic effects and inhibit carbohydrate digestive enzymes. Thus, this review demonstrates the therapeutic potential of NS that could be used as a complement or adjuvant for the management of DM and its complications. However, future research should be able to replicate and fill in the gaps of the study conducted to introduce NS safely to patients with DM.

## Introduction

Diabetes mellitus (DM) is one of the most prevalent metabolic disorders resulting from chronic hyperglycaemia due to problems in insulin secretion, insulin action, or both (1, 2). It affects people regardless of age, gender and race. The main consequence of DM development and the considerable morbidity and mortality it causes is the metabolic dysregulation of glucose homeostasis (3). The following classical classifications of DM were proposed by the American Diabetes Association in 1997: type 1 diabetes mellitus (T1DM), type 2 diabetes mellitus (T2DM), gestational DM, and specific types of diabetes due to other causes (4). T1DM and T2DM are the most common types of DM (2). Approximately 90–95% of those with DM develop T2DM (4). Global prevalence data from the International Diabetes Federation Atlas 2013 showed that the prevalence of DM in adults aged 20–79 years is 8.3% (382 million) of the worldwide population (2).

T2DM is characterized predominantly by insulin resistance with relative insulin deficiency, which increases the insulin demand of insulin-target tissues (4–6). Insulin levels may appear normal or elevated with normal β cell function, but their secretion is defective and insufficient to compensate for insulin resistance (5). People with T2DM are usually obese, and obesity is able to cause some degree of insulin resistance (5, 6). Additionally, those with an increased percentage of body fat distributed in the abdominal region are also affected by T2DM (5, 6). Nevertheless, diabetes-related health conditions can be prevented with pharmacological treatment and insulin. For example, metformin remains essential in treating T2DM, particularly for obese and overweight people (7). Metformin is necessary, as it increases insulin sensitivity and suppresses hepatic gluconeogenesis (8). Other therapeutic approaches include gene therapy, diabetic therapy targeting β cells and β cell regeneration, stem cells and enhancing the self-replication of β cells. Some of these therapeutic approaches are promising; however, their safety and effectiveness remain elusive (7).

**Figure 1:**
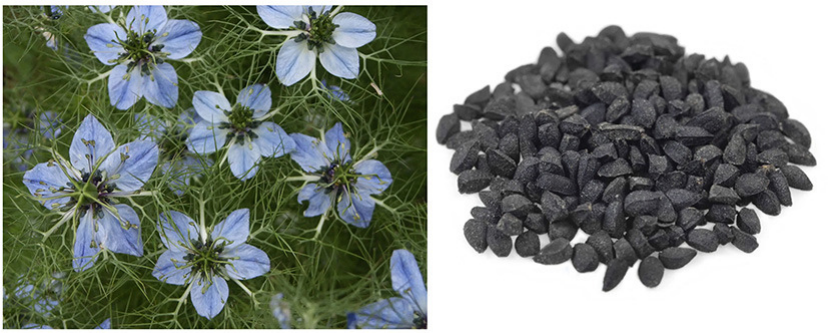
*Nigella sativa* plants and seeds.

Since ancient times, medicinal plants have been used and proven effective against diseases. These plants are believed to be effective and benefit physiological and pathological processes, as they can prevent, reduce or treat multiple diseases (9). *Nigella sativa* Linn. (NS; Figure 1) is an annual indigenous herbaceous plant that belongs to the buttercup family Ranunculaceae, a family of flowering plants, and the genus *Nigella* with 14 species, including *N. arvensis, N. ciliaris, N. damascene, N. hispanica, N. integrifolia, N. nigellastrum, N. orientalis*, and NS (10). Previously, NS is used only as a spice and in traditional medicine. However, nowadays, it is used extensively in the pharmaceutical and food industries (11).

NS constitutes a broad spectrum of therapeutic activities, including antidiabetic, diuretic, antihypertensive, anticancer, immunomodulatory, analgesic, antimicrobial, anthelmintic, anti-inflammatory, spasmolytic, bronchodilatory, gastroprotective, hepatoprotective, renal protective, antifungal, and antioxidant properties; increases maternal lactation and fertility and boosts the immune system (12, 13). NS's antidiabetic effects could improve clinical and glycaemic control in DM (14). In addition, numerous clinical studies have demonstrated the efficacy of NS on DM (15).

Several reviews have been conducted pertaining to the broad pharmacological effects of NS. However, very little literature has been done specifically on DM and its complications.

Thus, this review aims to gather and summarize up-to-date information on the potential health benefits of NS on DM and its complications from different animal models, clinical trials and *in vitro* studies. We also highlight the utilization of NS as the precursor to synthesize nanoparticles, which are beneficial in managing DM. Besides that, this review also specifies the different DM animal models and study designs, as well as the type of NS used in the study to perceive how NS influences the outcome in DM management. The findings could help provide a basis for developing new complementary antidiabetic medications with fewer side effects and excellent efficacy.

## Methods

A literature search was performed to identify and map out relevant articles related to the effects of NS on DM. Peer-reviewed, full-text English articles up to May 2022 were collected from electronic databases, including Scopus, MEDLINE *via* EBSCOhost and Google Scholar. The following set of keywords was used: (1) “*Nigella sativa*” or “*Nigella sativa* oil” or “*Nigella sativa* extracts” and (2) “diabetes mellitus.” The literature search was further supplemented by referencing related review articles and scientific reports found in the search results. All available *in vitro, in vivo* and human studies that reported the effects of NS or NS oil or NS seed on DM were included in this review. Meanwhile, studies on the effects of an individual bioactive component of NS, the mixture of NS with other herbs and studies on gestational diabetes (GDM) were excluded.

## Physicochemical and phytochemical contents of NS

NS is composed of remarkable levels of iron, copper, zinc, phosphorus, calcium, thiamin, niacin, pyridoxine, and folic acid (12, 16, 17). Additionally, it has a maximal nutritional value, including substantial amounts of vegetable protein (20–85%), fiber (7–94%), fat (38.02%), and carbohydrate (31.94%) (18). NS also has various amino acid components, including glutamate, arginine and aspartate, as well as cysteine and methionine (18). In addition, NS seeds contain 26–34% fixed oil from major fatty acids, such as linoleic acid (64.6%) and palmitic acid (20.4%) (17). Furthermore, NS seed oil comprises 0.4–2.5% essential oil (19).

Various factors can affect NS's phytochemical content, including the growing areas, maturation stage, processing procedures and isolation techniques (20). Using different extraction methods or solvents, some researchers also found different compounds that other studies have not report. For instance, in oil samples extracted with a cold press, hexane, tetrahydrofuran, ethanol, dichloromethane, and methanol produced various amounts of thymoquinone (21). Many studies have been conducted to determine the bioactive components of NS. Table 1 summarizes the various phytochemical compounds of NS that have been identified from different origins.

**Table 1:** Phytochemical compounds of NS from different origins.

**Origin**	**Type**	**Major phytochemical compounds**	**Potential activity**	**References**
Iran	Seed	α-thujene (PubChem-17868) α-pinene (PubChem-6654) sabinene (PubChem-18818) 3-carene (PubChem-26049) P-cymene (PubChem-7463) γ-terpinene (PubChem-7461) trans-sabinene hydrate (PubChem-6427504) thymoquinone (PubChem-10281) thymol (PubChem-6989) longifolene (PubChem-289151) palmatic acid (PubChem-985)	Antioxidant Antitumour	(22)
Turkey	Seed	α-thujene (PubChem-17868) o-cymene (PubChem-10703) limonene (PubChem-22311) γ-terpinene (PubChem-7461) terpinen-4-ol (PubChem-2724161) methyl chavicol (PubChem-66957732) tymoquinone (PubChem-10281) trans-sabynil acetate (PubChem-6430313) carvacrol (PubChem-10364) longifolene (PubChem-289151) (E)-caryophyllene (PubChem-5354499)	Antimicrobial Antituberculosis Antifungal	(23)
Tunisia	Seed, shoot, and root	p-cymene (PubChem-7463) α-thujene (PubChem-17868) α-pinene (PubChem-6654) β-pinene (PubChem-14896) limonene (PubChem-22311) γ-terpinene (PubChem-7461) gallic acid (PubChem-370) (–)-p-hydroxybenzoic acid (PubChem-135) chlorogenic acid (PubChem-1794427) vanillic acid (PubChem-8468) p-coumaric (PubChem-637542) ferulic acid (PubChem-445858) trans-2-hydroxycinnamic acid (PubChem-54693535) trans-cinnamic acid (PubChem-16213746) epicatechin (PubChem-72276) (+)-catechin (PubChem-73160) quercetin (PubChem-5280343) apigenin (PubChem-5280443) amentoflavone (PubChem-5281600) flavone (PubChem-10680)	Anticariogenic Antimutagenic	(24, 25)
Bangladesh	Seed	p-cymene (PubChem-7463) thymoquinone (PubChem-10281) α-thujene (PubChem-17868) carvacrol (PubChem-10364) β-pinene (PubChem-14896) limonene (PubChem-22311) methyl linoleate (PubChem-5284421) sabinene (PubChem-18818)	Not mentioned	(26)
India	Seed	p-cymene (PubChem-7463) α-thujene (PubChem-17868) thymoquinone (PubChem-10281) propyl-4- methyl-1-cyclohexene (PubChem-11572) sabinene (PubChem-18818) terpinen-4-ol (PubChem-2724161) trans-4-methoxy-thujane (PubChem-129845680) γ-terpinene (PubChem-7461)	Antibacterial Anticancer Antidiabetic Proapoptotic Antiproliferative	(26–29)
Poland	Seed	p-cymene (PubChem-7463) γ-terpinene (PubChem-7461) α-thujene (PubChem-17868) carvacrol (PubChem-10364) α-pinene (PubChem-6654) β-pinene (PubChem-14896)	Not mentioned	(30)

**Figure 2:**
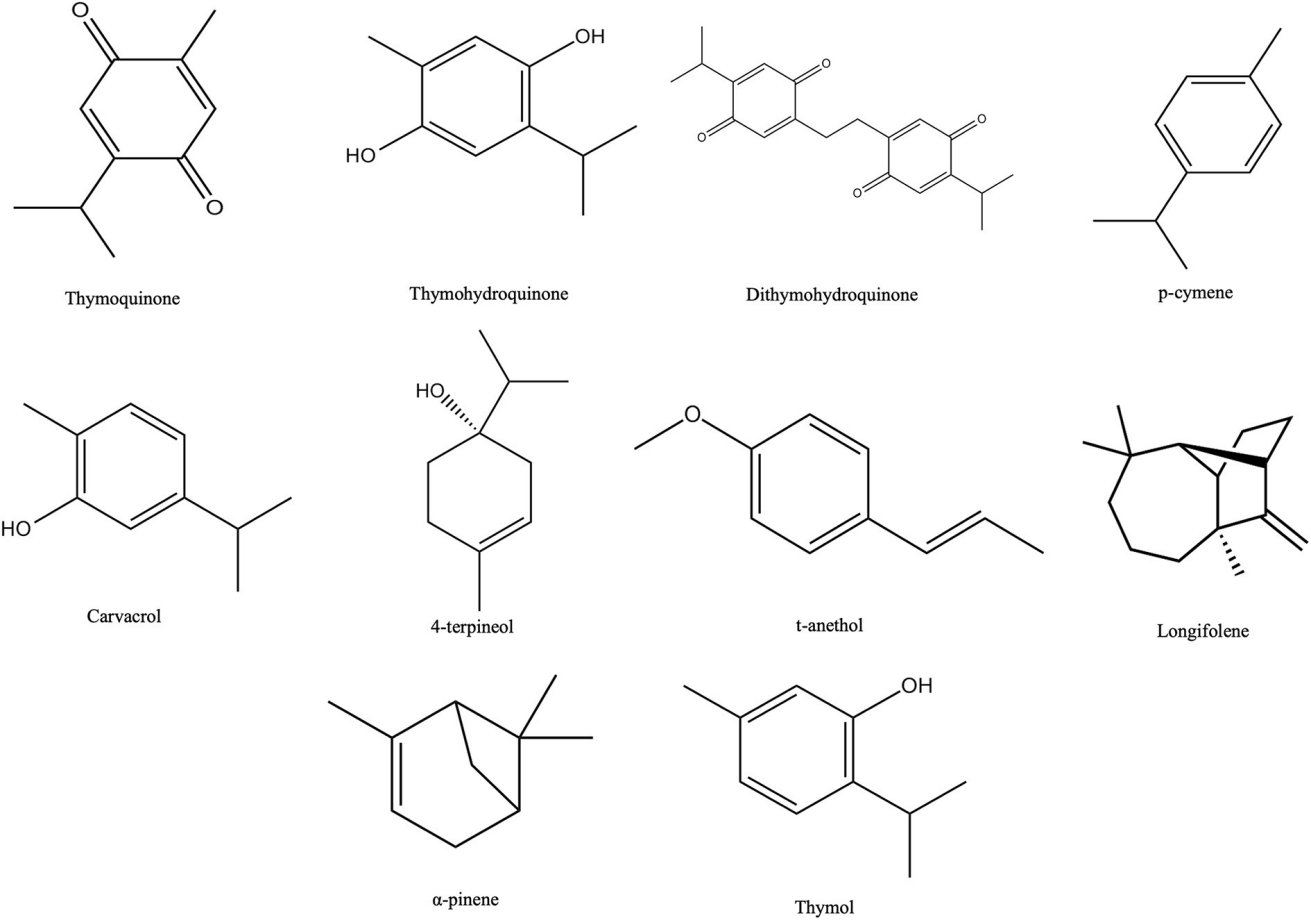
Chemical structures of major phytochemical compounds in NS.

Thymoquinone, thymohydroquinone, dithymoquinone, p-cymene, carvacrol, terpinen-4-ol, t-anethol, sesquiterpene longifolene, α-pinene, and thymol are the major phytochemical compounds found in NS (Figure 2) (12). Thymoquinone is the major active principle of NS oil and exhibits antidiabetic and antitumour activity against breast, lung, prostate, liver, colon and pancreatic cancers (31). Findings from previous studies revealed that thymoquinone accounts for 38–40% of total NS bioactive components (32). On the other hand, Singh et al. (33) reported p-cymene as the major component with 36.2% in the NS essential oil and linoleic acid as the major component with 53.60% using acetone extract. Besides these compounds, other compounds are found in trace amounts, such as camphene, sabinene, phenol, 2-methyl-5-(1-methyl ethyl), α-terpinene, β-terpinene, γ-terpinene, limonene, and many others.

## Effect of NS on DM

**Figure 3:**
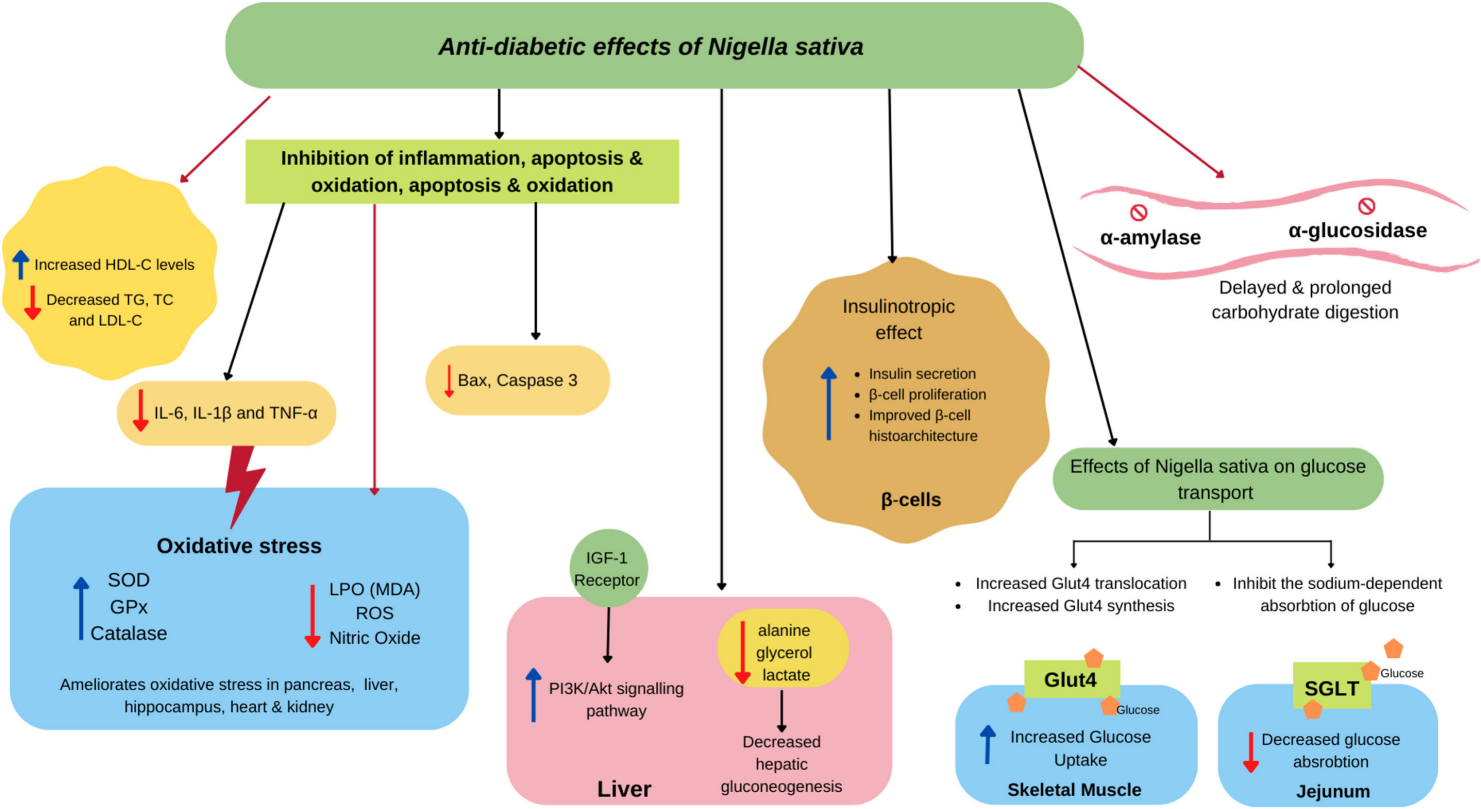
Proposed antidiabetic effects of NS. NS attenuates insulin secretion by ameliorating β cell proliferation and improves the structure of the islet of the pancreas. In the small intestine, NS agents delay the digestion and absorption of food because of the inhibitory activity of α-amylase and α-glucosidase. NS also modulate SGLT causing less glucose absorption in the jejunum epithelium and activating GLUT4, which is associated with increased glucose absorption in skeletal muscle. NS also alleviates insulin resistance by activating the PI3K/Akt signaling pathway, which promotes GLUT4 biogenesis and translocation. NS also decreases hepatic gluconeogenesis by reducing its precursors. Besides, NS exerts a potential in scavenging ${\text{O}}_{2^{-}}$, HO·, DPPH, and other free radicals to lighten oxidative stress, resulting in the less production of malondialdehyde (MDA) and nitric oxide. NS also increase the contents and activity of antioxidant enzymes GSHPx, SOD, and CAT. NS improves dyslipidaemia, which could reduce inflammatory markers, such as IL-6, IL-1β, and TNF-α, along with the reduction of apoptosis markers, such as Bax and caspase-3.

One of the most explored properties of NS is its antidiabetic potential. Studies involving different animal models, clinical trials, and *in vitro* studies have been conducted widely. This study reviewed 50 studies, including 31 animal studies, six *in vitro* studies, and 13 clinical trials. Both NS oil and extracts possess antidiabetic properties, as summarized in Table 2, and their proposed mechanism is illustrated in Figure 3.

**Table 2:** Summary of NS's effects on DM and its complications from *in vivo* and *in vitro* studies.

**Diabetic animal model/*in vitro***	**Treatment dosage**	**Duration of study**	**Outcomes of the study/major findings**	**References**
Female Wistar rats induced with 50 mg/kg STZ	0.2 mg/kg/day NS oil	30 days	↓ Blood glucose levels, serum total oxidant status (TOS) and MDA ↑ SOD and total antioxidant status (TAS)	(34)
Female Wistar rats induced with 45 mg/kg STZ	400 mg/kg NS oil	21 days	↓ Blood glucose levels; cardioprotective effect evidenced ↓ Myositis, hyaline degeneration, and Zenker's necrosis ↑ Bcl-2 expression	(35)
Adult female Wistar rats induced with 55 mg/kg STZ	2 mL/kg NS oil	10 weeks	↓ Albuminuria and the kidney weight/body weight ratio ↑ Podocin (podocyte-specific marker) ↓ Collagen IV, TGF-β1, and VEGF-A	(36)
Adult male Wistar rats induced with 60 mg/kg STZ	1 mL/kg NS oil	2 weeks	↓ Heart and brain nitric oxide (NO) and MDA ↑ GST, GSH, CAT, and serum CK-MB Restored norepinephrine, dopamine, and serotonin	(37)
Male Syrian hamsters induced with 230 mg/kg nicotinamide and 65 mg/kg STZ	400 mg/kg NS oil	4 weeks	↓ Blood glucose ↑ Insulin levels, ↑ pancreatic insulin immunoreactivity %	(38)
Hamster induced by 65 mg/kg STZ	400 mg/kg NS oil	4 weeks	↓ Blood glucose levels and HbA1c ↓ Glucose production in isolated hepatocytes in response to incubation with gluconeogenic precursors (alanine, glycerol, and lactate) ↑ Phagocytic activity and phagocytic index of peritoneal macrophages and lymphocyte count in peripheral blood	(39)
Wistar rats induced by 75 mg/kg STZ	400 mg/kg/day NS oil and TQ	6 weeks	↓ Blood glucose levels but not parallel by stimulation to an insulin release	(40)
Male Wistar albino rats treated with HFD for 2 weeks and 35 mg/kg STZ	2 mL/kg NS oil	21 days	↓ Brain TBARS, NO, and XO; ↑ GSH, GPx, GST, and SOD ↓ Sera and brain TNF-α, IL-6, IL-1β, iNOS NFκ-Bp65, and AGEs ↓ AChE (cholinergic function marker) ↓ Aβ-42 clearance by IDE ↑ GSK-3β level ↓ APP, BACE1, RAGEs, p53, and NF-κBp65 ↑ BDNF, SIRT1, ADAM10 gene expression ↑ IR, PI3K, IRS1-pTyr612, AKT1-pSer473, and GSK-3β-pSer9	(41)
Male albino Sprague–Dawley rats treated with HFD for 2 weeks and 35 mg/kg STZ	100 mg/kg NS oil	21 days	↑ Insulin receptor expression, Insulin like growth factor-1 (IGF-1) and phosphoinositide-3 kinase (PI3K) ↓ ADAM-17, blood glucose levels, lipid profile, TBARS, NO, serum insulin/insulin receptor ratio, and TNF-α	(42)
Adult male Wistar rat induced by 45 mg/kg STZ	2 mL/kg NS oil	30 days	↓ FBG ↑ Insulin levels, ↑ Pancreatic and hepatic CAT and GSH, ↑ Insulin immunoreactivity % and mean pancreatic islet diameter ↑ Lipid profile	(43)
New Zealand male rabbit induced with 150 mg/kg 10% alloxan	20 mL/kg NS seeds aqueous extract	2 months	↓ Glucose and MDA concentrations ↑ GSH and ceruloplasmin concentrations ↓ LPO-induced liver damage	(44)
Female Wistar albino rats induced with 50 mg/kg STZ	0.2 mL/kg NS volatile oil	4 weeks	↑ Insulin immunoreactive parts % and preservation of β-cell number moderate ↑ Increase in the lowered secretory vesicles with granules ↓ Destruction with loss of cristae within the mitochondria of β-cell	(45)
Male Wistar rats induced with 60 mg/kg STZ	100, 200 and 400 mg/kg NS seed ethanolic extracts	6 weeks	↓ Serum glucose and lipids ↑ eNOS ↓ VCAM-1 and LOX-1 ↓AGE and aldose reductase level	(46)
Male Wistar rats induced with 60 mg/kg STZ	200 and 400 mg/kg NS hydroalcoholic extracts	42 days	↓ Serum glucose levels ↓ MDA ↑ Thiol content of the hippocampus	(47)
Female Wistar rats induced with 200 mg/kg alloxan	0.2 g/kg NS aqueous extract	6–8 weeks	↓ Blood glucose levels ↑ Insulin level and HDL-C ↓ TGs, TC, LDL-C, and TBARS	(48)
Male Sprague–Dawley rats induced with 60 mg/kg STZ	4.0% fixed oil and 0.3% essential oil	8 weeks	↑ Hepatic SOD, CAT ↓ NO production ↑ Tocopherol content and expression of hepatic enzymes (glutathione peroxidase glutathione reductase and glutathione transferase)	(49)
Male Wistar rats induced with 60 mg/kg STZ	200 and 400 mg/kg NS hydroalcoholic extracts	6 weeks	↓ Blood glucose level, TC, TGs, and VLDL-C Improved the antioxidant status (↓ MDA and ↑ thiol, SOD and CAT of renal tissue)	(50)
Male Sprague–Dawley rats induced with 60 mg/kg STZ	4.0% fixed oil and 0.3% essential oil	8 weeks	↑ Insulin level and Total Antioxidant Capacity (TAC) ↓ Blood glucose level ↓ TC, LDL-C,TGs, and MDA	(51)
Female Wistar albino rats induced with 230 mg/kg nicotinamide and 65 mg/kg STZ	400 mg/kg NS seed extract and 400 mg/kg NS seed fat-free methanolic extract	30 days	↓α-glucosidase enzyme of two polar lipid fractions compared with acarbose	(52)
Sprague–Dawley rats induced with 50 mg/kg STZ	2 mL/kg 5% NS aqueous extract; 0.2 mL/kg, NS oil	30 days	↓ Serum glucose ↑ Serum insulin ↓ Pancreatic tissue MDA and ↑ SOD	(53)
Kunming mice treated with HFD for 4 weeks and a single injection of STZ	35, 70, and 140 mg/kg NSSP	4 weeks	↓ FBG, glycosylated serum protein, TGs, TC, LDL-C, MDA, TNF-α, IL-6, and IL-1β ↑ Insulin, HDL-C, TAC, SOD, and CAT ↑ Expression of p-AKT and GLUT4 of skeletal muscle	(54)
Male albino rats induced with 50 mg/kg STZ	200 mg/kg AgNPs prepared by NS extract 200 mg/kg	21 days	↓ Glucose, AGE, and aldose reductase ↑ Insulin ↓ TNF-α, NF-κB, and S100B ↓ MDA and NO ↑ SOD and GSH ↑ Nitrotyrosine ↑ TKr A	(55)
Male Sprague–Dawley rats induced with 65 mg/kg STZ	1,000 mg/kg/day Cold-pressed NS extract	8 weeks	↓ HbA1c concentration ↑ Capillary lumens ↓ Dermal capillary basement membrane thickening	(56)
Adult female Wistar rat induced with 50 mg/kg STZ	0.2 mg/kg/day NS oil	4 weeks	↓ Blood glucose level ↓ Bax and Caspase-3 expression in abdominal and thoracic aortic sections	(57)
Male Sprague–Dawley rats induced with 50 mg/kg STZ	2 mL/kg/day 5% NS aqueous extract 0.2 mL/kg/day NS oil and 5 mg/kg/day	4 weeks	↓ Pancreatic COX-2 enzyme, LPO, and MDA ↑ SOD	(58)
Meriones shawi given hypercaloric food	48 mg/kg/day of NS ethanolic extract	4 weeks	↓ Blood glucose levels ↑ Insulin levels and improve glucose tolerance in OGTT ↑ HDL-cholesterol ↓ Liver and skeletal muscle TGs contents ↑ Skeletal muscle and liver ACC phosphorylation ↑ Skeletal muscle GLUT4	(59)
Sprague–Dawley rats	2 g/kg/day aqueous extract of NS	6 weeks	Improved body weight ↑ Inhibition of sodium-dependent glucose transport across isolated rat jejunum ↑ Glucose tolerance as efficiently as metformin in OGTT	(60)
Male Wistar albino rats induced with 55 mg/kg STZ	300 mg/kg/day ethanol extract of NS	4 weeks	↓ Blood glucose levels ↑ Insulin levels ↑ TBARS, hydroperoxides, CAT, and SOD levels ↓ Glutathione and glutathione peroxidase in the liver and kidney	(61)
Male Wistar albino rats induced with 50 mg/kg STZ	0.2 mL/kg/day NS volatile oil	4 weeks	↓ Pancreatic tissue MDA, serum NO ↑ SOD, GSH-Px, and CAT ↑ Insulin immunoreactive β-cell	(62)
Male Wistar rats induced with 50 mg/kg STZ	0.2 mL/kg/day NS volatile oil	4 weeks	↓ Blood glucose levels ↑ Insulin levels ↑ Number of insulin-immunoreactive β-cells	(63)
Wistar albino rats induced with 150 mg/kg of alloxan (i.p.)	125 and 250 mg/kg/day NS ethanolic extracts	4 weeks	↓ Serum glucose and MDA levels ↑ SOD and GSHPx ↑ Diameter and amount of Langerhans islet cells ↑ Pancreas regeneration activity	(64)
Long Evans rats induced with 90 mg/kg STZ *in vitro*-isolated islets	NS seed methanolic extracts	*In vivo* and *in vitro*	↓ Post-prandial glucose and disaccharidase enzyme activity ↑ Glucose tolerance, GI motility and acute oral sucrose load assay Delayed glucose absorption	(65)
*In vitro*-isolated pancreatic islets from male Wistar albino rats	NS defatted and subfraction extracts	*In vitro*	↑ Glucose-induced insulin release from the pancreatic β-cells (highest in basic subfraction of the NS)	(66)
α-Glucosidase and α-amylase assay	NS seed extracts	*In vitro*	↓ Intestinal α-glucosidase and pancreatic α-amylase activity (highest in acetone fraction of the NS) The fraction with highest inhibitory activities are rich in apigenin and gallic acid	(67)
Albino mice and Wistar rats induced by 140 mg/kg alloxan	NS	*In vitro, in vivo*, and *in situ*	↓α-amylase activity and intestinal glucose absorption	(68)
α-Glucosidase and α-amylase assay	NS AuNPs	*In vitro*	↑ Antioxidant activity by DPPH assay ↓α-amylase and α-glucosidase activities	(27)
α-Glucosidase and α-amylase assay	NS AgNPs	*In vitro*	↑ Inhibition of carbohydrate-hydrolysing enzymes, such as α-amylase, α-glucosidase, and DPP-IV	(69)

### *In vitro* and *in vivo* studies

DM is a complex multifactorial metabolic disorder characterized by hyperglycaemia and lack of insulin release or insulin resistance. The pathophysiology of DM is closely associated with impaired redox homeostasis, activation of the inflammatory signaling cascade, and dyslipidaemia. Insulin, the key regulator hormone released by the β cell of the pancreas, is essential in maintaining glucose homeostasis. Hence, restoring insulin release and its sensitivity is warranted as one of the therapeutic strategies in managing DM.

Reports from recent studies have shown NS manage to alleviate the plasma glucose level due to its insulinotropic effects. In these studies, the proposed mechanisms for this effect are through the modulation of insulin receptor genes, increasing the insulin/insulin receptor ratio (42), enhancing the brain's insulin signaling pathway (41) and increased in insulin release from the increment of β cell activity (38).

The potential effect of NS in promoting insulin release has been studied extensively in T2DM animal models using a combination of streptozotocin (STZ) and nicotinamide or a combination of low-dose STZ with a high-fat diet (HFD). Rchid et al. further validated these findings in their *in vitro* study. They found that NS can stimulate glucose-induced insulin release, which is the highest in the basic fraction compared to the acidic and neutral fractions (66). The basic fraction of NS contains amino acids and some basic compounds, which could contribute to this particular effect of NS. This result signifies that the hypoglycaemic effect of NS is partially mediated by the potentiation of insulin release by the β cell.

Interestingly, El-Dakhakhny et al. reported that the hypoglycaemic effect of NS is not parallel to the stimulation of insulin release. In this study, they found that NS oil and its bioactive compounds, nigellone and thymoquinone, could stimulate insulin secretion in isolated rat pancreatic islets in the presence of glucose. However, as the insulin release does not reflect the blood glucose level, they postulate that NS possesses an extrapancreatic role in causing the hypoglycaemic effect. However, in this study, they used a much higher STZ (75 mg/kg) dose to induce the experimental animal, signifying more extensive damage to the β cell, which mimics T1DM rather than T2DM, as reported in the previous study (40).

Pancreatic tissue are vulnerable to diabetes-induced oxidative stress due to low expression of antioxidant enzyme and high production of endogenous ROS (70). This tissue damage could further impair the insulin released, deteriorating the diabetes condition. The effect of NS ameliorating insulin release from the β cell was further supported by the improvement in the β cell histoarchitecture findings. Studies revealed that administration of NS causes an increase in insulin immunoreactive percentage and mean pancreatic islet diameter, the ability to preserve the pancreatic β cell integrity, and increased area of insulin immunoreactive β cells in diabetic rats (43, 53). Additionally, Kanter et al. reported that treatment with 0.2 mL NS volatile oil for 4 weeks could lead to partial regeneration or proliferation of the pancreatic β cells in STZ-induced female diabetic rats (45). These improvements in β cell structure and functions could restore insulin release, which helps in overall blood glucose control in both T1DM and T2DM.

The hypoglycaemic effect seen in NS could also be attributed to the role of glucose transport. In a study by Meddah et al., 2 g/kg/day NS extract caused a reduction in glucose reabsorption from the intestine by modulating SGLT1, the primary glucose transporter in the intestine (60). The inhibition of SGLT1 not only improves glucose absorption but can also induce the release of incretin, such as glucagon-like peptide (GLP-1), which augments glucose-induced insulin secretion and prevents the release of glucagon (71). Thus, by acting on SGLT1, NS could help maintain blood glucose homeostasis. Normally, insulin facilitates glucose uptake in skeletal muscle and adipocytes through GLUT4 activity. The translocation of the GLUT4 to the cell surface helps in glucose transport into the cell, which reflects insulin sensitivity (72). In diabetes, the normal harmony between GLUT4 and insulin activity is disrupted. The prolonged hyperinsulinemia and hyperglycaemia worsen insulin resistance, which causes the glucose to be unable to enter the cell. Administration of 48 mg/kg/day of NS ethanolic extract in STZ-induced diabetes rats for 4 weeks shows upregulation in GLUT4 activity and synthesis in the skeletal muscle. This finding corresponds to the other parameter, such as improvement in blood glucose level, insulin and glucose tolerance following NS treatment.

#### Hypolipidemic effects

In diabetes, hyperglycaemia cause reduction in lipoprotein lipase activity which leads to hyperlipidaemia (73). Diabetic dyslipidaemia, characterized by increased fasting and post-prandial blood glucose, low-density lipoprotein (LDL-C), and decreased high-density lipoprotein (HDL-C), represents the major link with increased cardiovascular risk of diabetic patients (74). Fat accumulation has also been linked to systemic oxidative stress in animal and human studies due to the generation of inflammation and oxidative stress. NS has been shown to cause improvement in dyslipidaemia and high blood glucose levels in various diabetes studies. A study by Bensiameur-Touati et al. reported that the supplementation of 0.2 g/kg NS improved dyslipidaemia in diabetic rats by increasing the HDL-C and decreasing the triglycerides (TGs), cholesterol and low-density lipoprotein (LDL-C) (48). This finding is supported by Mohebbati et al. as they found administration of NS hydroalcoholic extracts for 6 weeks decreased TC, TGs, and VLDL-C (50). The essential fatty acid content in the NS itself could be among the factors that augment the improvement of dyslipidemia and its complications (75). Interestingly, utilization of NS seed polysaccharide also revealed similar improvement in lipid profile following treatment for 4 weeks in the T2DM rat model (54).

#### Antioxidants effects

Hyperglycaemia control remains one of the focal therapeutic strategies in managing DM. Uncontrol chronic hyperglycaemia triggers the overactivity of the NADPH oxidase enzyme, increases adipocyte expansion and mitochondrial production of the reactive oxygen species (ROS). When the production of ROS supersedes the antioxidant activity, it will result in the formation of oxidative stress (76).

A plethora of studies have proven NS's pharmacological potential is mainly due to its antioxidant property. As a potential source of antioxidants, NS is shown to cause a reduction in ROS formation and lipid peroxidation (LPO), evidenced by a decrease in its by-product, malondialdehyde (MDA). NS also upregulates the activity of the antioxidant enzymes such as catalase (CAT), superoxide dismutase (SOD), glutathione peroxidase (GPx), or molecules such as glutathione (GSH) in both *in vitro* and *in vivo* studies, which include both serum and the target tissue.

Seflek et al. reported treatment with 0.2 mg NS oil daily for 30 days caused a significant reduction in total oxidant status (TOS) and MDA and an increase in the antioxidant enzyme, SOD, along with total antioxidant status (TAS) in STZ-induced rats (34). Similar findings have been reported in a study on diabetic myopathy. They found that administration of 1 mL/kg NS oil restores the antioxidant enzyme such as CAT, GSH, GST, and cardiac enzyme CK-MB and reduces the MDA level, which signifies the improvement of the oxidant balance (37). Balbaa et al. further supported this finding in their study using the T2DM animal model, which they reported a significant reduction in brain oxidative stress evidenced by a decrease in TBARS whilst increasing the GSH, GPx, GST, and SOD (41). They also found the NS administration for 21 days able to deplete one of the crucial enzymes in generating oxidative stress, xanthine oxidase (XO). XO utilized xanthine and hypoxanthine as reducing substrates and causes electron reduction of an oxygen molecule to yield ROS such as superoxide and hydrogen peroxidase (77). Thus, ROS generation can be controlled and minimized by lowering the XO activity. Another critical finding in this study is the NS potential in reducing the advanced glycation end products (AGE). AGE formed by the non-enzymatic reaction secondary to chronic hyperglycaemia propagates the oxidative stress reaction by activation of NADPH oxidase enzyme and increases the drive of mitochondrial ROS production (78). Similarly, Abbasnezhad et al. also reported a reduction in AGE following treatment for 6 weeks in STZ-induced rats (46).

In a more recent study, Alkhalaf et al. utilized the NS extract to synthesize the silver nanoparticles (AgNPs) and reported their antioxidant potential in reducing of both AGE and MDA (55). The study that ran for 21 days in STZ-induced rats also reported an increase in the antioxidant enzymes, SOD and GSH along with an increase in nitrotyrosine. The liver and kidney also benefit from the antioxidant effects of the NS as they cause an increase in TBARS, hydroperoxides, CAT and SOD levels and reduced glutathione and glutathione peroxidase following treatment with 300 mg/kg/day ethanol extract of NS for 4 weeks (61). A similar antioxidant potential is reported in the NS gold nanoparticle, which increased the antioxidant activity in the DPPH assay (27).

A comparison study between NS oil and the NS aqueous on the pancreas reported that both NS preparation protects against oxidative stress by decreasing COX-2, LPO, and MDA and increasing the antioxidant enzyme SOD, hence, providing pancreatic-protective effects. However, treatment with NS oil could not ameliorate the serum glucose level to normal value despite restoring the insulin level in diabetic rats (58). In another study, hydroalcoholic extracts of NS with doses of 200 and 400 mg/kg for 42 days were shown to improve oxidative status by increasing the thiol content and decreasing the MDA level in the hippocampus of diabetic rats (47).

#### Anti-inflammatory effects

Diabetes induced-oxidative damage may create ripple effects that trigger widespread inflammatory reactions, which further exacerbate the damage to the tissues. Studies have shown that the pro-inflammatory molecules such as interleukin 6 (IL-6), interleukin-1beta (IL-1β), and tumor necrosis factor (TNF-α) are significantly elevated in diabetic humans and experimental models (79). Furthermore, these pro-inflammatory markers serve a crucial role in metabolic inflammation caused by the impairment of serine phosphorylation of insulin receptor substrate 1 (IRS-1), which regulates insulin receptor signaling and consequently modulates insulin action (80).

Studies have reported that IL-1β, IL-6, and TNF-α were significantly reduced in the serum of HFD-STZ-induced diabetic rats treated with NS seed polysaccharides (NSSP) for 4 weeks (54). This may relate to the NS potential to significantly downregulate the gene and protein expression of inflammatory chemokines, as well as repress the activation of NFκB and MAPK pathways, which are crucial for cellular inflammatory responses (76). The green synthesis of silver nanoparticles (SeNPs) using NS seed extract also revealed similar effects in causing a reduction in the TNF-α and NFκB in diabetic nephropathy animal models (55). The generation of the massive pro-inflammatory cytokines further exacerbates the inflammatory reaction, which could potentiate insulin resistance by altering the insulin signaling pathways. NS administration was shown to reverse these effects by upregulating the gene expression of insulin growth factor 1 (IGF-1) and phosphoinositide-3 kinase (PI3K) (42). The increase in IGF-1 and PI3K helps promote insulin sensitivity which is crucial in maintaining blood glucose control, particularly in T2DM.

Vascular inflammation is another critical issue, which causes a high chance of morbidity and mortality in diabetic patients. Insulin resistance disrupted nitric oxide synthesis by reducing the activity of endothelial NO synthase (eNOS) along with increased production of a vasoconstrictor such as endothelin-1 (81). NS was found to reverse this effect by increasing the eNOS, which helps improve endothelial dysfunction (46). They also reported that NS treatment with ethanolic extracts for 6 weeks caused a reduction in vascular cell adhesion molecule 1 (VCAM-1), mainly used to allow firm adhesion of leukocytes to endothelial cells and promotes vascular inflammation (82). A previous study reported that NS bioactive compound thymoquinone disrupts the link between TNF- α signaling pathway that regulates the ICAM/VCAM expression in arthritis *in vitro* (83). This means that NS mediates its effects by modulating the adhesion molecule as well as controlling the inflammatory markers.

Interestingly, Balbaa et al. reported that the combination of NS oil and hypoglycaemic drugs such as metformin and glibenclamide inhibited the inducible nitric oxide synthase (iNOS) and NO formation in the brain tissue (41). As iNOS is considered one of the important regulators for insulin resistance, its inhibition minimizes the risk of disease progression. In an earlier study, Balbaa et al. also revealed a reduced serum NO in T2DM rats (42). Similarly, Hamdy and Taha also reported a significant reduction in NO levels in both heart and brain samples (37). Nitric oxide works like double edge swords, yielding different outcomes in various studies. The lack of NO leads to vascular damage by causing endothelial dysfunction, as mentioned earlier, and NO in excess causes the propagation of oxidative stress and inflammation. Hence, the NS's ability to restore NO homeostasis is warranted in managing DM.

#### Anti-apoptotic effects

The abundance generation of ROS and pro-inflammatory cytokines opens the gate for the activation of the apoptosis cascade and leakage of cytochrome-c, which lead to more detrimental effects. The extrinsic apoptotic pathway is initiated by binding the Fas ligand to the death receptor to activate the caspase complex. Whereas, the intrinsic apoptotic pathway is derived from the regulated B-cell lymphoma (BCL-2), which expresses both pro- and anti-apoptotic signals (84). In response to apoptotic stimuli, upregulation of BCL-2-associated X protein (BAX) and downregulation of BCL2 and has reported promoting cell death in diabetic models (85).

NS significantly increases the expression BCL2 in cardiac tissue following NS oil administration for 21 days (35). Cuce et al. also found a similar result as they reported a reduction in BAX and caspase 3 expression in the abdominal and thoracic aortic section, preventing apoptosis in the vessels of STZ-induced diabetic rats (57). The antiapoptotic effect of NS is not only beneficial for preventing cellular damage in DM but also resourceful in managing cancerous cells, as reported widely (86). These studies highly suggest that NS treatment could modulate both the intrinsic and extrinsic pathways of hyperglycaemia-induced cell death.

#### Hepatoprotective effects

Hyperglycaemia and insulin resistance in DM affect the carbohydrates, fats and protein metabolism, potentially leading to the formation of non-alcoholic fatty liver disease. If there is no proper intervention, there is a risk of progression to hepatitis, cirrhosis, and eventually hepatocellular carcinoma (87). Among the main triggers responsible for this deteriorating effect are oxidative stress and liver inflammation. Various studies have reported that high lipid peroxidation (LPO) could damage the hepatocyte, characterized by raised liver enzyme and alteration of the histoarchitecture of the liver (78). Administration of the NS oil for 30 days improved hepatic antioxidant enzymes such as CAT and GSH while preventing the LPO in the liver of STZ-induced rats (43).

Similar hepatoprotective effects were shown by Kaleem et al. using NS ethanol extract treatment for 4 weeks (61). They found that 300 mg/kg NS ethanol extract improved the antioxidant enzymes, such as CAT, SOD, and LPO products [thiobarbituric acid reactive substance (TBARS) and hydroperoxides] and reduced glutathione (GSH) and glutathione peroxidase (GSHPx) in both liver and kidney (61) of STZ-induced rats. Interestingly, a study on alloxan-induced rabbits also demonstrated improved GSH and ceruloplasmin concentrations whilst preventing LPO-induced liver damage in diabetic rabbits (44).

The hepatoprotective effect of NS is not limited to its antioxidant properties as they are also shown to improve the hepatic glycogen content (43). Furthermore, they postulated that the improvement of the hepatic glycogen content is due to the advancement of insulin secretion, which promotes glycogenesis in the liver.

Fararh et al. supported this finding and reported NS oil extracts could decrease hepatic gluconeogenesis by reducing its precursors (alanine, glycerol, and lactate) (39) and prevent the formation of glucose.

Interestingly, Sobhi et al. reported that treatment with NS seed extract did not show a hepatoprotective effect on acetaminophen following treatment for 4 weeks (52). Acetaminophen is an analgesic and antipyretic agent with a direct hepatotoxic effect in a large dose. Even though NS did not possess a protective effect on the liver from acetaminophen, no hepatotoxicity was found when tested alone in STZ-NA-induced rats. The liver plays a huge role in maintaining blood glucose levels; therefore, preserving its function is warranted as a treatment modality for DM.

#### Digestive enzyme inhibition

Another issue faced in T2DM patients is a spike in blood sugar levels following a meal, referred to as postprandial blood glucose. Hydrolysis of carbohydrates by a digestive enzyme, such as α-amylase and α-glucosidase, contributes to the increase of blood glucose levels in the body (88). Therefore, one therapeutic strategy aimed at maintaining the blood glucose level is inhibiting these digestive enzymes. Currently, a few inhibitors are available in the market, such as voglibose and acarbose. However, the issue with these medications is the disturbing side effect, such as flatulence and digestive disorders. NS's role as an inhibitor carbohydrate digestive enzyme was studied in 2019 by Hannan et al. (65). The methanolic extract of NS causes a reduction or delaying carbohydrate digestion and absorption by improving glucose tolerance and reducing disaccharidase enzyme activity in STZ-induced diabetic rats (65).

Tiji et al. also reported similar findings. NS extraction reveals to cause a reduction in both intestinal α-glucosidase and pancreatic α-amylase activity, with NS acetone extract showing the greatest inhibitory effect. This suggests the possibility of the polyphenol compounds, such as catechin and gallic acid, as the main contributors to digestive enzyme inhibitory effects (67). A different finding is reported by Dalli et al. as they found that the *in vitro* α-amylase percentage of inhibition is greatest in the aqueous fraction (92.24%), which is similar to the positive control used in this study, Acarbose; followed by methanol (86%), ethanol (81%), dichloromethane and n-hexane fractions; both are showing 64% inhibition. *In vivo* assessment of the α-amylase inhibition also revealed that aqueous and an ethanolic fractions of NS reduced the glucose 30 min after the oral sucrose load. However, in the *in situ* glucose absorption, methanolic extract caused better control compared to the aqueous fraction. The various degrees of inhibition in different fractions suggest that the α-amylase inhibitory effect of NS is attributed to different bioactive compounds such as quercetin, kaempferol, thymoquinone and others, as shown in their HPLC and GC-MS findings (68).

Interestingly, the NS digestive enzyme inhibition was also evaluated using different preparations of nanoparticles.

Due to its high antioxidant potential, NS has been utilized as a capping or reducing agent in producing nanoparticles. Vijayakumar et al. reported that the silver nanoparticles synthesized using NS (NS AgNPs) possessed a high degree of inhibition in both α-amylase and α-glucosidase enzymes. They also reported an increase in the inhibition of the dipeptylpeptidase-IV enzyme (DPP-IV). DPP-IV acts by inhibiting the incretins such as GLP-1 and GIP. As the incretins stimulate insulin release, inactivation of the incretins by the DPP-IV enzyme exerts a negative response toward high blood glucose control, especially in T2DM. Inhibition of this DPP-IV enzyme could further potentiate better blood glucose control, as reported in this study. It can work similarly to gliptin, the available DPP-IV inhibitor in the market (69). In a more recent study, Veeramani et al. reported that NS-coated gold nanoparticles (AuNPs) show potent antioxidant activity in *in vitro* DPPH assay, as well as causing significant inhibition on α-amylase and α-glucosidase in a dose-dependent manner (27). Similarly, the microencapsulation of the hydroacetone extract of NS also reported a high α-amylase enzyme inhibition compared to acarbose, signifying that the microencapsulation of NS enhances the inhibitory effect against the carbohydrate digestive enzyme (89).

### Clinical trials

Due to the promising NS antidiabetic potential shown in preclinical studies, clinical trials have been conducted to translate the findings into clinical use. In this review, 13 clinical trials were selected, as summarized in Table 3. However, in these clinical studies, patients are on the standard regime of an oral hypoglycaemic agent (OHA) or other medications (antihypertensive and antihyperlipidemic agents).

**Table 3:** Summary of NS's effects on DM from clinical trials.

**Study design**	**Dosage**	**Duration of study**	**Outcomes of the study**	**References**
Interventional study (pre–post study) 41 patients with T2DM	0.7 g/day NS oil	80 days	↓ FBG ↑ Insulin and AST levels	(90)
Interventional study (pre–post study) 94 patients with T2DM, three groups	1, 2, and 3 g/day powdered NS	12 weeks	2 g/day dosage caused ↓ FBG, 2 h post-prandial glucose and HbA1c	(91)
Interventional study (pre–post study) 66 T2DM, 2 groups (*n* = 41, 25)	5 g/day NS aqueous extract (NS tea)	6 months	↓ FBG, 2 h post-prandial glucose, HbA1c, AST, ALT, total serum bilirubin, creatinine, and blood urea	(92)
Randomized, double-blinded control trial 70 patients with T2DM, 2 groups (*n* = 35)	5 mL/day NS oil	1 year	↓ FBG, 2 h post-prandial glucose, HbA1c, and body weight	(93)
Randomized, single-blinded control trial 114 patients with T2DM, 2 groups (*n* = 57)	2 g/day powdered NS	1 year	↓ FBG, insulin resistance, HbA1c, and TBARS ↑ TAC, SOD, and GSH Improved β cell activity	(94)
Randomized, single-blinded control trial 60 patients with T2DM, 2 groups (*n* = 30)	2 g/day powdered NS	1 year	↓ HbA1c Protects the heart from diastolic dysfunction Improves left ventricle systolic function	(95)
Randomized, double-blind, placebo-controlled clinical trial 43 patients with T2DM (*n* = 20, 23)	500 mg/day NS oil	8 weeks	↓ FBG, TC, LDL-C, TGs, BMI, and blood pressure (SBP and DBP)	(96)
Prospective, open-label randomized clinical trial at outpatient endocrinology clinic 44 newly diagnosed patients with T2DM (*n* = 23, 21)	450 mg/3 times per day NS oil	3 months	↓ Body weight, weight circumference, and BMI ↑ Fasting insulin; HDL-C and TAC ↓ ALT; TC, LDL-C, TGs	(97)
Double-blind randomized clinical trial study 50 patients with T2DM	1,000 mg NS oil in two capsules	8 weeks	↓ FBG, TGs, TC, LDL-C, serum CRP, and MDA ↑ Serum HDL-C	(98)
Prospective, open-label, second-phase trial 94 patients with T2DM, three groups	1, 2, and 3 g/day powdered NS	12 weeks	2 g/day NS displayed significant ↓ TC, TGs and LDL-C and significant ↑ HDL-C/LDL-C.	(99)
Single-blind, non-randomized 114 patients with T2DM, 2 groups (*n* = 57)	2 g/day powdered NS	1 year	↓ SBP, DBP, MAP, TC, LDL-C, TC/HDL-C ratio, and LDL-C/HDL-C ratio	(100)
Randomized control trial 68 patients with T2DM, 2 groups (*n* = 34)	2.5 mL/day NS oil	12 weeks	↓ Blood glucose, serum creatinine, blood urea and 24 h total urinary protein levels ↑ Glomerular filtration rate, 24 h total urinary volume and hemoglobin level	(101)
Randomized, double-blind, placebo-controlled trial 72 patients with T2DM, 2 groups (*n* = 36)	3 g/day NS oil	12 weeks	↓ Fasting blood sugar, HbA1c, TGs, LDL-C, insulin resistance ↑ Insulin and HDL-C	(102)

Similar to *in vitro* and *in vivo* studies, the clinical trials revealed that NS has the potential as a supplementary treatment for T2DM. The duration used for the clinical trials ranges from 8 weeks (96, 98) up to 1 year (93–95). The treatment of T2DM for 8 weeks with NS oil has shown marked improvement in terms of glycaemic control (decreased in FBG and HbA1c and increased insulin level) and the amelioration of the lipid profile (considerable reduction in T-cholesterol, LDL and TG and an increase in HDL). The improvement in these parameters could help minimize the cardiometabolic risk factors in T2DM (96, 98). Furthermore, hyperlipidaemia is considered as one of the important triggers of the oxidative inflammatory cascade in T2DM (76); therefore, NS's hypolipidaemic property could reduce the risk of T2DM progression and complications (98–100).

Also, ameliorating dyslipidaemia could help improve cardiovascular function in patients with T2DM. Hadi et al. further reported that along with the improvement in lipid profile, patients had shown a substantial decrease in BMI, waist circumference, systolic blood pressure (SBP) and diastolic blood pressure (DBP) at a dose of 500 mg NS daily (96). This finding is supported further by Bamosa et al., who found that treatment with 2 g powdered NS for a year could reduce blood pressure, protect the heart from diastolic dysfunction and improve left ventricular (LV) systolic function (95). In this study, an echocardiogram was performed at baseline and repeated at 6 and 12 months follow-ups to evaluate the cardiac function of patients with T2DM. They found that NS could preserve diastolic function by monitoring the transmitral flow ratio, which was substantially reduced in the placebo group. In addition, NS also protects the heart from the development of LV hypertrophy and improves systolic function.

The cardioprotective effect of NS was consistent with the animal studies, which showed a reduction in oxidative stress parameters in the aorta and cardiac tissue following the treatment with hydroalcoholic extract of NS for 6 weeks in STZ-induced diabetic rats (47). This result suggests that ameliorating oxidative stress is one of the NS's potential actions in causing improvement in cardiac function in DM. This antioxidant effect is further supported by Kaatabi et al., who reported a considerable increase in total antioxidant capacity (TAC), CAT, GSH and SOD and a remarkable reduction in TBARS following treatment with 500 mg NS powdered capsule in T2DM patients (94). This finding is consistent with a study by Kooshki et al., which showed a reduction in serum MDA, the LPO by-product, in patients with T2DM treated with 1,000 mg NS for 8 weeks (98). The improvement in antioxidant enzymes could help minimize oxidative stress progression in T2DM and could be a mechanism contributing to the hypoglycaemic effect of NS.

Kaatabi et al. also reported increased pancreatic β cell activity with considerably lower insulin resistance after a year of NS treatment (94). A similar finding was reported by Bamosa et al. who demonstrated that treatment with NS oil for 12 weeks helps in the reduction of insulin resistance calculated by HOMA-IR and the improvement of β cell function in a non-controlled trial study (91). Another study reported that the effects of NS on fasting insulin and insulin resistance in newly diagnosed patients with T2DM were comparable to the standard oral hypoglycaemic agent (OHA) metformin following treatment for 3 months (97). However, different studies reported that NS causes a reduction in FBG, but no remarkable differences in insulin secretion and HOMA-IR after adjusting for changes in body weight, dietary intake and baseline value (102). This result suggests that the clinical study outcome is heavily influenced by the dosage, duration, type of clinical study, dietary intake, baseline glucose, ethnicity and activity.

The improvement in pancreatic β-cell function has also reported widely in *in vitro* (66) and *in vivo* diabetes studies (38, 41, 43, 45, 53, 62, 63). The amelioration of β-cell function could explain the improvement of insulin levels and the overall fasting and post-prandial blood glucose levels in clinical trials (91–93). The improvement in post-prandial blood glucose could be attributed partially to the inhibition of the carbohydrate digestion enzyme by NS (52, 67, 68). Post-prandial blood glucose control helps prevent the post-meal spike in blood glucose levels, which is crucial in preventing T2DM progression and complications. After NS treatment, improvement in glycated hemoglobin levels resulted a well-controlled blood glucose level (91–95, 102). The organ-protecting effect was also observed in the liver and kidney, in which treatment with NS caused a reduction in serum creatinine and urea with improvement in glomerular filtration rate and 24 h urinary volume (101) and a reduction in ALT (90), AST and total serum bilirubin (92).

## Discussion

NS belongs to the genus Nigella in the family Ranunculaceae. Some of the plants belonging to this genus include *N. damascene, N. arvensis, N. hispanica, N. nigellastrum* and *N. orientalis*. Compared to the other plants in the same genus, only NS has reported antidiabetic properties. However, phytochemical evaluations of these plants show that they possess bioactive compounds, which could be beneficial in managing DM, such as quercetin, quercitrin, hyperoside, carvacrol, thymohydroquinone, and kaempferol, to name a few (103). These compounds have been studied extensively and show potential antioxidant, anti-inflammatory and antiapoptotic properties. *N. damascene*, one of the plants from the same genus, was reported to have antioxidant effects owing to its quercetin and its derivative content. The polar extract of NS was also shown to possess a wide range of phenolic compounds, which were found to have high antioxidant capacity. However, the potential effect of nigella genus plants is not only limited to the antioxidant activity as they are also reported to ameliorate dyslipidaemia. Kokdil et al. reported the administration of *N. orientalis* and *N. segetalis* fixed oils orally (1 mL/kg/day) on Wistar Kyoto rats for 4 weeks increased HDL-C and *N. orientalis* also caused a decrease in LDL-C and VLDL-C. Both plants also increased the total antioxidant status in the experimental rats (104). Due to the high potential of NS, the other plant of the same genus should be explored to unravel their antidiabetic potential further.

The *in vivo* and *in vitro* trials have shown a wide array of NS potential to be utilized as hypoglycaemic agents. Some NS actions considered the main attributes of its antidiabetic potentials are antioxidant, anti-inflammatory and insulin-mimetic properties. Additionally, the action of NS in lowering blood glucose levels mimics the currently available OHAs in the market. For instance, similar to Metformin, NS is shown to activate the AMPK pathway (59), enhance insulin sensitivity and glucose uptake and fatty acid oxidation, and reduce intestinal glucose absorption (60). Not only that, NS has proven potential in the modulation of the carbohydrate digestive enzyme to prevent and control the fluctuations of postprandial blood glucose level, similar to the actions of miglitol, voglibose, and acarbose, currently available antidiabetic drugs that are prescribed to T2DM patients. Other than its individual effects, NS could potentiate the effect of these antidiabetic drugs, and synergise the effect, which could help in more holistic control in managing the blood glucose level. Furthermore, the dosage of the medication and the toxicity could be reduced when applied in combination, hence improving the patients' overall outcome.

Based on the clinical trials, several DM biomarkers could be assessed to trace the effects of NS and its bioactive compound in causing the desired effect. The potential biosensing marker could be the basis of DM parameters such as fasting and post-prandial blood glucose level and glycated hemoglobin; to evaluate the long-term blood glucose control as well as compliance toward the treatment and the plasma insulin level. Additionally, the cardiometabolic biosensing marker, such as lipid profile, could be monitored to prevent progression toward diabetic cardiomyopathy (105). However, it should not be left unnoticed that in these clinical trials, patients are still on the OHA's, which means the effects seen in these patients are synergistic effects with the medication they took rather than individual effects of NS. Future studies must be conducted to evaluate the NS effects in the combination of OHA and its pharmacological and toxicological profile for future clinical applications.

However, despite numerous studies showing NS promising results to be utilized in DM management, it is still bound to several limitations that require attention before clinical application. Similar to other plant resources, the NS's bioavailability in terms of solubility, stability and half-life remains the main issue in elucidating the warranted effects. Modulation of the structure and different doses and strategies have been studied extensively in preclinical settings, which gives a wide array of information before safely being conducted in the clinical trial. Additionally, the drug-herb interaction is another important issue to be further validated to ensure clinical therapeutic efficacy. It may cause risks of unwanted pharmacokinetic and pharmacodynamic interactions when co-administered. Hence, a more structured and extensive human trial is needed to further verify the mechanism of action, pharmacological effect, the range of safe doses and toxicological profile before utilizing NS as an antidiabetic agent.

## Conclusion

This review article demonstrates the therapeutic potential of NS could be used as a complement or adjuvant for the management of DM and its complications. Various preparations of NS have been studied to provide therapeutic strategies for maximizing the antidiabetic effects of NS. NS exhibits multifactorial activities that target several tissues at the same time owing to its phytochemical properties. The proposed mechanism includes ameliorating oxidative, inflammatory, apoptotic, and insulinotropic effects and inhibiting carbohydrate-digesting enzymes, to name a few. Similar promising hypoglycaemic effects have been found in clinical studies involving real patients with T2DM, further validating the potential of NS. As a whole, NS appears to play a role as a complement or adjuvant in managing DM and its complications. However, future research should be able to replicate and fill in the gaps to introduce NS safely in patients with DM.

## Author contributions

MM, SA, and MK: conceptualization, methodology, and writing—review and editing. SA, NM, EA, and MA: writing—original draft preparation. SA and EA: visualization. All authors have read and agreed to the published version of the manuscript.

## Conflict of interest

The authors declare that the research was conducted in the absence of any commercial or financial relationships that could be construed as a potential conflict of interest.

## Publisher's note

All claims expressed in this article are solely those of the authors and do not necessarily represent those of their affiliated organizations, or those of the publisher, the editors and the reviewers. Any product that may be evaluated in this article, or claim that may be made by its manufacturer, is not guaranteed or endorsed by the publisher.
